# Recommending 24-hour attendant care: A qualitative study exploring the clinical decision-making process of occupational therapists in Ontario, Canada

**DOI:** 10.1177/02692155251336574

**Published:** 2025-04-27

**Authors:** Bao-Zhu Stephanie Long, Kishana Balakrishnar, Maryna Mazur, Elana Maria, Kathleen Hennessy, Mathew Rose, Behdin Nowrouzi-Kia

**Affiliations:** 1 Department of Occupational Science and Occupational Therapy, Temerty Faculty of Medicine, 7938 University of Toronto, Toronto, Ontario, Canada; 2 Matthew Rose & Associates Medical Legal Consultants, Toronto, Ontario, Canada; 3 Krembil Research Institute-University Health Network, Toronto, Ontario, Canada; 4 Centre for Research in Occupational Safety & Health, Laurentian University, Sudbury, Ontario, Canada; 5 Centre for Addiction and Mental Health, Toronto, Ontario, Canada

**Keywords:** 24-Hour attendant care, occupational therapy, decision-making, qualitative research

## Abstract

**Objective:**

This study aims to explore how occupational therapists working in private practices in Canada use clinical indicators and tools to determine if clients require 24-hour attendant care.

**Design:**

A qualitative research study.

**Setting:**

The setting involved semi-structured, one-on-one interviews with occupational therapists in Canada.

**Participants:**

Occupational therapists were selected through purposive sampling: (1) registered Canadian occupational therapists, (2) with over 10 years of private practice experience, and (3) who have assessed the need for 24-hour attendant care at least once before the study.

**Main measures:**

The interviews were conducted, transcribed, coded, and thematically analyzed by two researchers using Braun and Clarke's protocol. The paper is also reported based on the consolidated criteria for reporting qualitative research guidance.

**Results:**

The study involved nine occupational therapists (eight women and one man), with 14 to 24 years of private practice experience in Ontario. Three main themes in the decision-making process for 24-hour attendant care were identified: (1) Individualized and Holistic Assessments; (2) Clinical Expertise-Based Decision-making; and (3) Risk Assessment in Decision-Making.

**Conclusions:**

This study provides a greater understanding of the decision-making process of occupational therapists working in Canada when recommending 24-hour attendant care. However, further research and development of guidelines are needed to support occupational therapists in this area.

## Introduction

Canadian occupational therapists in private practice play a key role in assessing patients’ need for 24-hour attendant care, which involves continuous supervision and various services (e.g., custodial, medical, personal, or rehabilitative services) seven days a week.^1^ The recommendation for 24-hour care has significant long-term implications for patients, often based on a single assessment.^2^ However, the lack of established guidelines limits therapists’ clinical reasoning and decision-making.^3^ This is especially critical in the auto insurance sector, where assessments involve individuals with severe, long-term disabilities (e.g., traumatic brain injuries, spinal cord damage) from motor vehicle accidents.^4,5^ Accurate, consistent assessments are essential for both patient welfare and financial accountability,^6^ as without clear frameworks, there may be under- or overestimations affecting safety.

Research on occupational therapists’ role in assessing patients’ needs has increased, but few studies focus on their decision-making process for recommending 24-hour care. A scoping review by Maranan and Rose found limited literature on the topic and highlighted inconsistencies in the areas of assessment, variability in legal testimony quality, and growing demand for occupational therapists’ expertise.^1^ This points to the need for clearer frameworks to support consistent recommendations. Further research by Flemming et al.^3^ revealed that, while common assessments like MoCA and Berg Balance Scale are widely used, therapists employ 166 different tools, illustrating variability in practice. The urgency of addressing this issue is heightened by an aging population and increasing demand for long-term care services,^7^ placing greater pressure on occupational therapists to make high-stakes decisions regarding 24-hour care recommendations. Additionally, ongoing policy shifts in healthcare funding and home care services are influencing access to resources,^8^ making it even more critical to establish consistent and evidence-based decision-making processes. These findings emphasize the need for better understanding of how therapists interpret assessment results, select tools, and build confidence in their decisions.

Recognizing the significance of this issue and the absence of qualitative studies examining the clinical reasoning processes of occupational therapists in Canada, this qualitative study seeks to explore how Canadian occupational therapists in private practice determine the need for 24-hour attendant care for patients. By conducting in-depth interviews, the study will address the research question: *How do Canadian occupational therapists working in private practice use clinical indicators and tools to determine if 24-hour attendant care services are required*? This study will examine the factors that influence occupational therapists’ assessment decisions, the reasoning behind their interpretation and use of assessment results, the key information sources they rely on, and the elements that impact their confidence in making recommendations, with the goal of further informing the development of standardized frameworks to improve care quality and consistency in Canadian occupational therapy.

## Methods

### Study design

The research employed a qualitative study design with a descriptive approach to investigate how occupational therapists make decisions regarding the necessity of 24-hour attendant care. This study received approval from the University of Toronto's ethics review board (Human Protocol Number: 45294), and all participants gave both written and verbal consent to participate before interviews began. Semi-structured one-on-one interviews were carried out, which are particularly effective for collecting qualitative data, as they allow for a deeper understanding of individuals’ experiences and perceptions.^9^ Interviews were conducted between January and March 2024. This paper follows the guidelines outlined in the consolidated criteria for reporting qualitative research (COREQ) (see COREQ Checklist in Supplementary Materials).^10^

### Research team

The interviews for this study were conducted by EM and KH, two female second-year learners of the Master of Science in Occupational Therapy Program at the University of Toronto. All participants were provided with a letter of information and a consent form to participate in the study, outlining the research objectives. Participants were informed that the researchers were conducting this study to understand how occupational therapists make decisions regarding 24-hour attendant care to work towards establishing standardized guidelines for occupational therapists in this area. Authors B-ZSL and KB supported the reviewing of transcripts to identify recurring themes and ensure the thoroughness and consistency of the analysis. Authors MR and BN-K provided supervision throughout the entirety of the study, guiding the conceptualization, data collection, analysis, and manuscript writing phases, due to their prior experience in qualitative research and clinical backgrounds as occupational therapists.^11,12^

### Study population/participants

This study's population consisted of occupational therapists currently practicing in a private practice setting in Canada. The inclusion criteria encompassed: (1) experienced registered Canadian occupational therapists who are currently practicing in any province, (2) occupational therapists who have had more than ten years of experience working in private practice, and (3) occupational therapists who have assessed the need for 24-hour attendant care at least once prior to the study. Non-English-speaking occupational therapists were excluded. The initial list of participants was derived from Author MR's professional network. However, some participants declined and suggested others with relevant knowledge and expertise in the field. Purposive sampling was then conducted before sending invitations to ensure that participants met specific criteria. OTs were selected based on a review of previous cases and their professional experience, including years of practice, courtroom testimony (indicating respected expertise in the OT profession), and experience working with both defense and plaintiff cases. This approach ensured that participants had the necessary background in private practice and familiarity with 24-hour care assessment, increasing the relevance of their insights.

Fifteen selected occupational therapists were invited via email; however, five declined due to scheduling conflicts, and one withdrew before the interview phase because of retirement plans and personal time constraints. Consequently, nine occupational therapists from Ontario participated in the study. Despite efforts to recruit participants from across Canada, jurisdictional differences posed challenges. Lower litigation rates, smaller occupational therapy populations, and fewer therapists with experience recommending 24-hour supervision in other provinces made recruitment difficult. Since Ontario has the largest population of therapists and the highest litigation rates, the geographic focus naturally narrowed to Ontario. This number was deemed sufficient to achieve data saturation, as previous qualitative research suggests that rich and meaningful themes can emerge with sample sizes ranging from five to 30 participants per group,^13,14^ though no fixed rule exists for determining an exact sample size.^15,16^

### Data collection

Before meeting with the participants, basic demographic information was collected for each individual, including their registration number (a unique identifier provided by the relevant provincial college), to verify their occupational therapy registration. This information was gathered through a survey created in REDCap, with a link sent to each participant. The survey asked participants about key details related to their work as occupational therapists (e.g., years of practice, the number of attendant care decisions they have made). For more information, refer to the REDCap Demographic Survey Questions within the Supplementary Materials.

A pre-established interview guide was created by the entire research term used to direct each interview (refer to Interview Questions within the Supplementary Materials). Participants did not receive the interview guide before the interview. Not providing participants with the interview guide beforehand is a deliberate approach to ensure that their responses are spontaneous and reflect their authentic thoughts, rather than being influenced by prior reflection on the questions. This method helps to capture more genuine and unbiased data, as it reduces the risk of participants tailoring their answers based on preconceived ideas about the topics. Participants were included in a single interview. The interview questions were created using the Person-Environment-Occupation model.^17^ They focused on three key areas: (1) psychosocial factors in decision-making, (2) decisions regarding 24-hour supervision on occupational injuries (e.g., orthopedic, neurological) and environmental limitations, and (3) litigation considerations. Psychosocial factors in decision-making relate to individualized assessments by considering how personal circumstances and mental health impact choices, ensuring decisions are tailored to the person's needs. Decisions regarding 24-hour supervision and environmental limitations connect to evidence-based decision-making by emphasizing the need to evaluate work environments, accessibility, and support systems when determining the best course of action. Litigation considerations are linked to risk assessment in decision-making, as legal factors and potential consequences influence how risks are evaluated in the context of returning to work or ongoing disability.

All interviews were conducted and recorded on Zoom and lasted for 60 minutes long. Interviews were conducted in a setting the participant chose, typically their workplace or home. The interviews were audio recorded and transcribed. To deepen understanding of the data, authors EM and KH independently listened to and reviewed each transcript for accuracy several times before the analysis. Transcripts were analyzed throughout the study to monitor for saturation. Data saturation was assessed by reviewing transcripts after each interview to determine whether new themes or insights were emerging. Authors EM and KH independently reviewed transcripts and compared findings, discussing whether additional interviews were contributing novel information. Once multiple consecutive interviews yielded no new codes or themes, data collection was considered complete. However, transcripts were not returned to participants for feedback.

### Data analysis

All collected data were securely de-identified and stored on a University of Toronto-protected SharePoint system. Interview recordings were transcribed using Microsoft Word's transcription feature and verified by the research team. Specifically, to ensure transcription accuracy, the research team rigorously reviewed each automated transcript by cross-referencing it with the audio recordings and correcting any discrepancies. This approach enabled transcription-based analysis and enhanced the study's integrity.^18^ Finalized transcripts underwent a member-checking process, where participants were allotted a two-week period to review and provide feedback on their transcripts. This step further increased validity, allowing participants to clarify responses and confirm the accuracy of conveyed meanings.^19^ The descriptive data was then thematically coded following recommendations in accordance with Braun and Clarke's (2006) framework.^20^

This analysis employed an inductive approach, where themes were developed directly from the data, allowing for a more open exploration without predefined categories or theories. Specifically, two independent coders, EM and KH, reviewed the transcripts to identify recurring themes, with support from authors B-ZSL and KB, along with supervisors MR and BN-K.^21^ The quality of the analysis was ensured through coding until data saturation was reached, marked by the absence of new codes emerging.^22^ Once saturation was achieved, the codebook was finalized to ensure consistency in the final analysis. Subsequently, both coders re-coded the transcripts collaboratively, achieving consensus on all identified codes. All transcripts were then recoded using NVivo software (Version 14; QSR International),^23^ with the finalized codebook guiding the process. An audit trail was meticulously maintained throughout coding to mitigate potential biases and strengthen study trustworthiness.^24^ Through in-depth discussions between the authors, each code was scrutinized to derive and refine key themes.^21^

## Results

**Figure 1.**
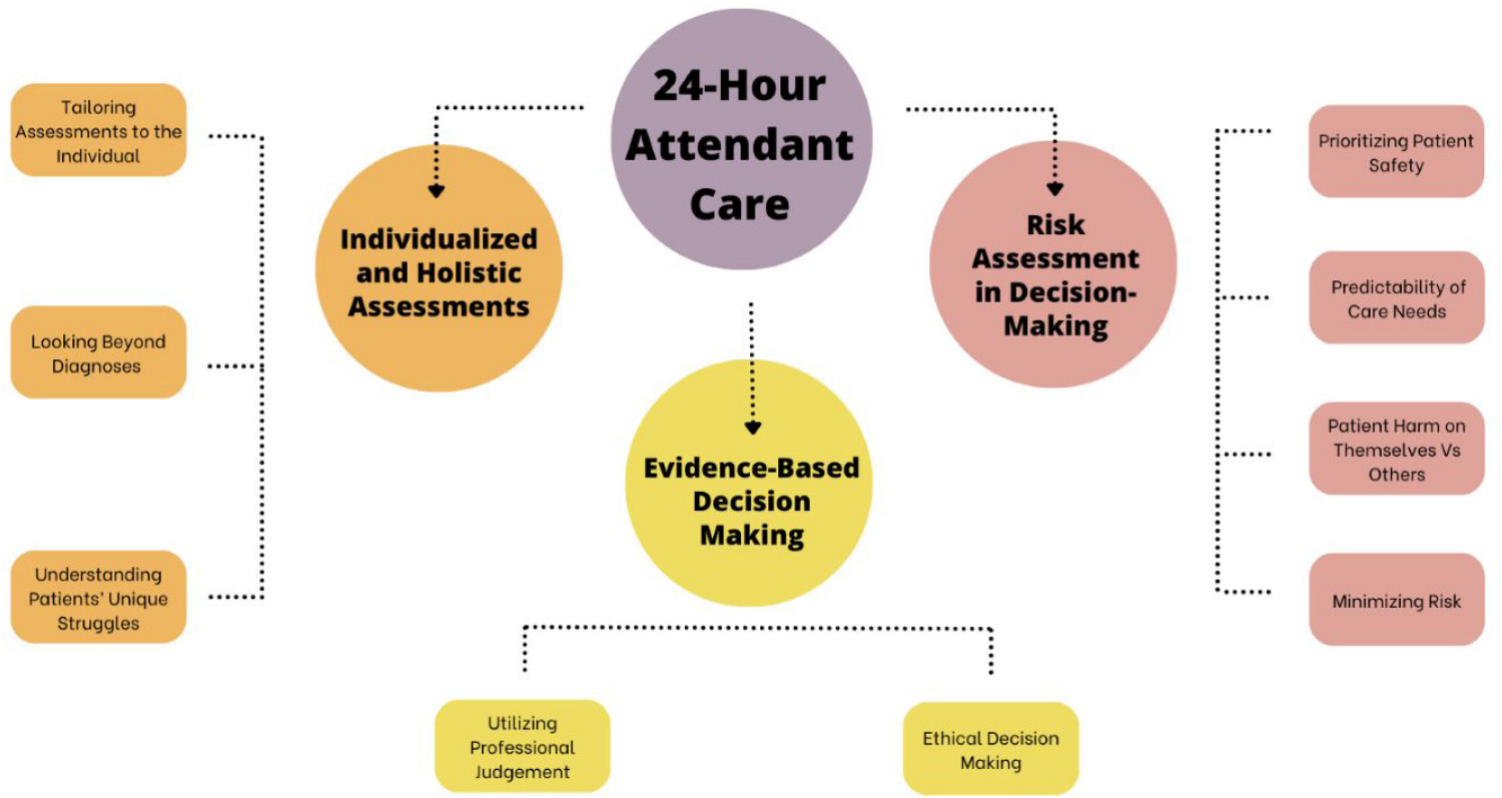
Concept map of identified themes on 24-hour attendant care based on clinical indicators from occupational therapists.

Nine interviews were conducted with occupational therapists (eight females, one male) aged between 37 and 67 years, all practicing in Ontario, with each interviewee having at least ten years of experience in private practice. Detailed demographic information for the participants is shown in Table 1. Three overarching themes were collated among the qualitative interviews: (1) Individualized and Holistic Assessments; (2) Clinical Expertise-Based Decision Making; and (3) Risk Assessment in Decision-Making (see Figure 1).

**Table 1.** Participant demographic information.

Participant no.	Years of experience as an occupational therapist	Years working in private practice	Working sector (public vs private)	Number of evaluations where 24-hour care was deemed necessary	Number of recommendations involved in legal proceedings
1	24	14	Private	<25	Not specified
2	14	14	Private	≥100	5–10
3	24	11	Private	≥25 and <99	4
4	29	24	Both	≥100	Most recommendations
5	22	22	Private	≥25 and <99	Many recommendations
6	31	Not specified	Private	≥25 and <99	Not specified
7	24	21	Private	<25	4
8	45	23	Private	≥100	Not specified
9	25	21	Private	25	None

### Theme 1: Individualized and holistic assessments

Throughout the interviews, participants emphasized the necessity of tailoring assessments to the individual, collecting and integrating data from various sources to form a comprehensive picture of the client's overall functioning. This data collection goes far beyond simply looking at diagnoses or results of standardized assessment scores. Therapists endorsed gathering information through various methods, including in-depth client interviews, input from family and caregivers, observations of functional tasks, standardized testing, and review and consideration of available file information. One participant articulated the importance of this approach by stating that they do not determine levels of supervision solely based on a diagnosis. Instead, they focus on understanding the client's struggles and how they can manage their needs or seek assistance. This perspective reinforces that assessments should prioritize the individual's unique challenges and capabilities by considering their specific functional limitations, personal strengths, and coping strategies. By focusing on these aspects, therapists can tailor interventions and support plans that empower clients to manage their needs more effectively and navigate challenges with appropriate assistance.

- I don't look at [a] diagnosis and then determine that you need 24-hour supervision. What is it that you suffer from? And what is it that you aren't able to do for yourself or get that assistance in a way that you're capable of managing your own needs?……….But it is very functional. So I am asking questions not only what you do. What do you need? But what are you doing currently and and how are you sleeping and what happened the last time? I will try and kind of narrow it down. You remember the last time that you XYZ. What did you do then? You know. And then I'm able to kind of build a case whether it's for 24 h [care] or against [it]. (Participant 7)
- I typically do [a] review of course (the clinical notes and records), the medical file very well before seeing the client. So that's always something that I do first. Understanding the diagnosis and the nature of the injuries or the disabilities before seeing the patient is also very important because understand, you know, I mean I don't think I know everything. (Participant 5)
- I will always try and get collateral information from family members or friends or, you know, depending who they live with or who knew them before the accident and how things are changed. Or if they're just coming out of hospital, I'm doing that interview with hospital staff. (Participant 1)

All participants mentioned the need for flexibility and adaptability in their assessments, based on conversations with and observations of the client, to ensure assessments accurately reflect the client's unique needs and circumstances.

- Of course, this depends on the nature of the injuries. So I mean if I am evaluating someone and I know that there is, there are absolutely no physical impairments, but this person had a severe traumatic brain injury then obviously the focus of my testing is going to be geared more towards some cognitive testing methods. (Participant 5)
- Again, depending on who they are and whatever, but I will typically start with something fairly open-ended about emergencies in general and if I'm not loving what I'm hearing, then I start to close it down and I start to really sort of peck away at at specific problems that I might see until we're real specific and and that's how I might satisfy whether you know they are able to respond in an emergency from a clinical perspective. (Participant 7)

A client may seem functional in a controlled setting but struggle with real-life tasks, suggesting that flexibility in assessment is crucial. One therapist illustrated this point with an example: despite potentially scoring well on cognitive tests, a client might encounter difficulties with practical tasks, such as operating the latch on her bedroom door. This situation underscores that traditional assessments can overlook significant challenges, highlighting the importance of adapting approaches to capture a more comprehensive understanding of a client's capabilities and experiences.

- I could have done lots of cognitive tests and went ‘she's pretty intact.’ Until you give her a novel thing like the latch on her bedroom door. I'm sure she'd been out it before, but.. she physically got to the door, and she couldn't get out. (Participant 1)

### Theme 2: Clinical expertise-based decision making

The evaluative and analytical processes occupational therapists employ support evidence-based decisions on the appropriate intensity and duration of client care. Therapists emphasized the critical need to assess how specific evidence components may interact and compound, ultimately affecting a client's functional capabilities. All therapists underscored the pivotal role of professional judgment, which is cultivated through extensive experience and engagement with complex cases and diverse clientele, in guiding their decision-making processes.

- Part of this is definitely you go by gut feel too. You have to have that clinical sense and gut feel. (Participant 8)
- If you don't feel it's absolutely imminent, you know I don't know if you're going to get into cognitive things as well. You know, people with head injuries and all that. And is there judgment bad and when is their judgment off? That's a whole other. Uh. Think but it's experience and it's your clinical judgment. (Participant 2)

Many participants also noted an ethical or moral factor in their decision-making process, especially when dealing with pressures from the system to make decisions that align with their referral source (either plaintiff or defense). These therapists stressed the importance of making decisions that they feel ethically comfortable with, often reflecting on the impact these decisions have and the weight they carry.

- I can't say there is a hard and fast rule. These are clinical judgments we make, and my threshold is can I sleep at night having made this decision? Honestly, it sounds crazy, but that's sometimes what it comes down to. (Participant 8)
- So sometimes you end up recommending attendant care. Because you have someone within a household and are they safe within the household? Like if somebody becomes violent and there's other people within the household? Like children. Then you have to kind of separate the parent and then they still need 24 h supervision. Because people live in the context of their life. (Participant 9)

### Theme 3: Risk assessment in decision-making

Prioritizing client safety emerged as a crucial factor impacting the decision-making process. All therapists consistently emphasized the importance of assessing for and factoring both the imminence of risk for the client and the predictability of care needs. This included evaluating the risk of the clients harming themselves and the risk of them harming others, as well as the ability to determine future harm risks. One therapist described arriving for an assessment and having to terminate the assessment due to imminent risk for the client due to a deteriorating health condition.

- Sometimes in those situations, it isn't just about getting the report done, it's about keeping people alive. (Participant 9)
- I don't remember specifics, but I would recommend 24 h if I really felt someone was at imminent risk and I have medical foundation to support it. And if I didn't, then I wouldn't. So if somebody said Ohh they were, you know always at high risk and the accident has something nothing to do with it then it's hard to justify. (Participant 1)

Similarly, many therapists discussed the importance of keeping safety the main priority, minimizing risk as much as possible, and emphasizing that they prioritize safety above other clinical considerations. Completing a risk analysis was mentioned, but it's important to note that they do not use a formal tool to assess overall current or future risk. For example, one participant said they tend to side with the client when risk is too complex to determine. This focus on safety ensures that clients’ well-being is always a top priority in the decision-making process.

- I err on the side of caution. I'm not going to be someone who says I'm an expert in this and I think your risk is 0.4% and that's not worth 24/7. I'm probably going to go on the side of caution and say you should have access to someone. (Participant 2)
- It's based on safety for the person, whether it's your physical safety or emotional safety. And again, I always go back to if it were my family member would I leave them alone. What do I think that they need? And that to me is more of an indicator as to whether I would, would or would not give 24 h care based on what's in the documentation what I see, what they've said in collateral. (Participant 6)

## Discussion

This study identified three themes when providing valuable insights into occupational therapists’ decision-making process for 24-hour attendant care needs. Firstly, comprehensive, individualized, and holistic assessments that collect data from multiple sources are needed to make these determinations. Weighing the collected data also relies heavily on clinical expertise-based decision-making (e.g., personal skills, experience, knowledge, and ethical considerations). Finally, assessing for and mitigating risk is at the forefront of these decisions but lacks formal tools to determine its totality.

The challenges identified in this study are consistent with previous studies. In a European survey, accessibility to services is hindered by a shortage of practitioners in primary care, which affects the thoroughness of assessments needed for such complex decisions.^25^ This shortage is attributed to a lack of professional development opportunities, limited public and professional awareness of occupational therapy services, and insufficient research. For instance, countries like Germany, Spain, Italy, Austria, and Belgium have relatively high numbers of occupational therapists in private and independent practice, enabling more comprehensive assessments, while other countries like Georgia and Bulgaria lack practitioners in these settings,^25^ which could impede the availability of services necessary to determine 24-hour attendant care needs.

This study emphasized the importance of holistic and individualized assessments for determining the need for 24-hour attendant care, reflecting the profession's longstanding commitment to personalized, client-centered care,^26,27^ which has been shown to improve patient outcomes.^28–31^ Integrating data from various sources, such as client interviews, collateral input, functional observations, and standardized assessments, enables therapists to gain a comprehensive understanding of a client's functional abilities and needs.

Functional tasks that might be observed during an assessment include everyday activities like meal preparation, dressing, bathing, or managing personal hygiene.^32^ These tasks provide insight into the practical challenges a client faces in their daily life. In terms of standardized testing, therapists may use tools such as the Functional Independence Measure, which assesses the degree of a person's disability and how much assistance they require for activities of daily living.^33^ Other assessments, like the Montreal Cognitive Assessment MoCA, may be used to assess cognitive functions, which can be particularly important for clients with brain injuries or cognitive impairments.^34^ These standardized tests, alongside functional task observations, help therapists determine the level of care required, whether it 's 24-hour supervision or less intensive support.

This approach aligns with best practices in occupational therapy, which advocate for gathering relevant information through interviews with the client and family members^3^ and using standardized evaluations that assess both mental and physical performance.^35,36^ However, this process also requires occupational therapists to be highly skilled in selecting and interpreting assessment results, as these tools can vary depending on the clinician's training, knowledge, and context of practice.^37^ For instance, many therapists in the study reported receiving additional professional development to improve their proficiency with specific assessments and practice areas, highlighting the diversity in approaches and training within the profession.^3^ Given that no formal requirements exist for the quality or quantity of data collected, the decision-making process is influenced by factors such as the clinician's training, experience, and personal values.

The reliance on professional judgment and ethical considerations highlights the complex decision-making process for determining 24-hour supervision. Participants weighed evidence using their clinical experience and knowledge while striving to remain objective and truthful in their findings. However, they acknowledged facing pressures from funding and referral sources, which could affect their caseload and reputation within the community.^38^ Personal experiences influencing decisions are subjective and prone to biases, such as cognitive and affective biases, which can impact decision consistency and quality.^39^ Standardized guidelines could help mitigate these biases and improve decision-making.

Furthermore, risk assessment is central to clinical decision-making, as it helps identify potential hazards and guide interventions to reduce risk.^40^ Participants in this study expressed concerns about the lack of formal tools for assessing risk, pointing out that the absence of standardized frameworks for quantifying risk could lead to inconsistencies in decision-making and make it more difficult to defend their decisions in legal contexts. Exploring and implementing standardized risk assessment tools could improve decision-making consistency and provide a clearer framework for determining acceptable levels of risk.

This study has several limitations. All participants were Ontario-based occupational therapists, despite efforts to recruit from other provinces, which may limit the ability to capture regional, legal, or cultural differences. However, the issues explored in this study have broader implications, as they are relevant to occupational therapy practices in other regions and countries. Additionally, the small sample size of nine therapists affects the generalizability of the findings. Future research should include larger and more diverse samples to improve the validity and applicability of the findings. While the selected quotes were the most relevant and representative of the data, they reflect a more general understanding of the topic. Studies could aim to explore this topic further by encouraging more detailed responses. There is a need for further investigation into the clinical threshold for determining the need for 24-hour care. Specifically, it is essential to explore whether factors such as permanent disability or other clinical parameters should guide decision-making. Additionally, further education and training for occupational therapists on risk analysis and assessment is a critical factor in improving this process. These findings could help future practitioners in making more informed and consistent decisions amongst other practitioners, ultimately improving client outcomes.

The study emphasizes the importance of individualized, holistic assessments in determining 24-hour attendant care needs, with occupational therapists relying on comprehensive data, clinical expertise, and professional judgment. Risk assessment remains central to decision-making, with safety being prioritized, and the implementation of standardized assessment tools enhancing consistency and rigor in evaluating client needs.

**Clinical messages**

- Occupational therapists must conduct individualized assessments using diverse data sources—client interviews, functional observations, and standardized tools.
- Standardized risk assessments ensure consistency, defensibility, and alignment across healthcare settings.
- Staying up to date with the latest knowledge through continuing professional medical/health education is key to making evidence-based and client-specific decisions.

## Supplemental Material

sj-pdf-1-cre-10.1177_02692155251336574 - Supplemental material for Recommending 24-hour attendant care: A qualitative study exploring the clinical decision-making process of occupational therapists in Ontario, Canada Supplemental material, sj-pdf-1-cre-10.1177_02692155251336574 for Recommending 24-hour attendant care: A qualitative study exploring the clinical decision-making process of occupational therapists in Ontario, Canada by Bao-Zhu Stephanie Long, Kishana Balakrishnar, Maryna Mazur, Elana Maria, Kathleen Hennessy, Mathew Rose and Behdin Nowrouzi-Kia in Clinical Rehabilitation
