# Supplementary material for: Recommending 24-hour attendant care: A qualitative study exploring the clinical decision-making process of occupational therapists in Ontario, Canada
Source: Clin Rehabil. 2025 Apr 27;39(6):819–29. doi: 10.1177/02692155251336574 (PMC12141767; doi:10.1177/02692155251336574)
Supplement: sj-pdf-1-cre-10.1177_02692155251336574 - Supplemental material for Recommending 24-hour attendant care: A qualitative study exploring the clinical decision-making process of occupational therapists in Ontario, Canada [file sj-pdf-1-cre-10.1177_02692155251336574.pdf]

Consolidated criteria for reporting qualitative studies (COREQ): 32-item checklist

Developed from:

Tong A, Sainsbury P, Craig J. Consolidated criteria for reporting qualitative research (COREQ): a 32-item checklist for interviews and focus groups. *International Journal for Quality in Health Care*. 2007. Volume 19, Number 6: pp. 349 – 357

No. Item	Guide questions/description	Reported on Page #
**Domain 1: Research team and reflexivity**		
Personal Characteristics		
1. Interviewer/facilitator	Which author/s conducted the interview or focus group?	3
2. Credentials	What were the researcher's credentials? E.g. PhD, MD	3
3. Occupation	What was their occupation at the time of the study?	3
4. Gender	Was the researcher male or female?	3
5. Experience and training	What experience or training did the researcher have?	3
Relationship with participants		
6. Relationship established	Was a relationship established prior to study commencement?	3
7. Participant knowledge of the interviewer	What did the participants know about the researcher? e.g. personal goals, reasons for doing the research	3
8. Interviewer characteristics	What characteristics were reported about the interviewer/facilitator? e.g. Bias, assumptions, reasons and interests in the research topic	3
**Domain 2: study design**		
Theoretical framework		
9. Methodological orientation and Theory	What methodological orientation was stated to underpin the study? e.g. grounded theory, discourse analysis, ethnography, phenomenology, content analysis	2
Participant selection		
10. Sampling	How were participants selected? e.g. purposive, convenience, consecutive, snowball	4
11. Method of approach	How were participants approached? e.g. face-to-face, telephone, mail, email	4
12. Sample size	How many participants were in the study?	4, 7
13. Non-participation	How many people refused to participate or dropped out? Reasons?	4
Setting		
14. Setting of data collection	Where was the data collected? e.g. home, clinic, workplace	6

15. Presence of non-participants	Was anyone else present besides the participants and researchers?	N/A
16. Description of sample	What are the important characteristics of the sample? e.g. demographic data, date	Table 1
Data collection		
17. Interview guide	Were questions, prompts, guides provided by the authors? Was it pilot tested?	5
18. Repeat interviews	Were repeat interviews carried out? If yes, how many?	N/A
19. Audio/visual recording	Did the research use audio or visual recording to collect the data?	6
20. Field notes	Were field notes made during and/or after the interview or focus group?	6
21. Duration	What was the duration of the interviews or focus group?	6
22. Data saturation	Was data saturation discussed?	6
23. Transcripts returned	Were transcripts returned to participants for comment and/or correction?	6
**Domain 3: analysis and findings**		
Data analysis		
24. Number of data coders	How many data coders coded the data?	7
25. Description of the coding tree	Did authors provide a description of the coding tree?	Figure 1
26. Derivation of themes	Were themes identified in advance or derived from the data?	7
27. Software	What software, if applicable, was used to manage the data?	7
28. Participant checking	Did participants provide feedback on the findings?	6
Reporting		
29. Quotations presented	Were participant quotations presented to illustrate the themes/findings? Was each quotation identified? e.g. participant number	10-14
30. Data and findings consistent	Was there consistency between the data presented and the findings?	14
31. Clarity of major themes	Were major themes clearly presented in the findings?	9-14
32. Clarity of minor themes	Is there a description of diverse cases or discussion of minor themes?	Figure 1

REDCap Demographic Survey Questions

1. Age
2. Gender
    a. M, F, Other
1. How many years have you been practicing as an occupational therapist?
2. How long have you been working in private practice?
3. What area of occupational therapy do you work in? Instead- Do you work in a privately or publically funded practice or both. For example, OHIP.
4. How many times have you determined that a client was in need of 24-hour supervision/care?
5. Roughly, how many of your recommendations have been involved in legal proceedings?

Interview Questions

1) What clinical research do you rely on in making clinical decisions?
    a. What specific articles do you rely on?

2) What questionnaires / clinical testing have you utilized?
    a. Are there certain parts or sections of the questionnaires you particularly focus on to make a determination on 24-hour attendant care supervision?
    b. How do you weigh the evidence?

3) Have you made a determination based purely on psychosocial factors Ex: suicidal ideation?
    a. What clinical indicators or information would you rely on to determine a client requires 24-hour supervision based on psychosocial factors?
    b. What questionnaires would you utilize?
    c. How do you determine level of risk that future harm, such as suicidal ideation, is of high enough risk that 24-hour supervision is indicated?
    d. What is that threshold of risk that you use to determine 24-hour supervision?
    e. What level of risk would you consider sub-threshold or would indicate that 24-hour supervision is not required?
    f. How do you differentiate between the immediate need for hospitalization (emergency / psychiatric ward) and the need for 24-hour supervision in the home setting?
    g. Please describe a specific scenario where you did recommend 24 hour supervision and one where you did not recommend 24 hour supervision? Please explain differences in the scenarios and contrast?

4) Have you made a decision about a client's need for 24-hour supervision based purely on Orthopaedic injuries?
    a. How do you determine if the severity of injuries warrants 24-hour care?
    b. What is the threshold of determining 24-hour supervision?
    c. Please explain a scenario where you did AND did not recommend 24-hour supervision?

5) Have you made a decision about a client's need for 24-hour supervision based purely on Neurological injuries?
    a. How do you determine if the severity of injuries warrants 24-hour care?
    b. What is the threshold of determining 24-hour supervision?
    c. Please explain a scenario where you did AND did not recommend 24 hour supervision?

6) Have you made a decision that a client requires 24-hour supervision based on environmental limitations?
    a. Do you rely on egress times (time to exit building)?
    b. How do you measure this time? What factors do you consider?

c. What mitigating factors do you consider in determining 24 hour supervision for 24 hour
d. How do you factor in things like lifelines, emergency response plans, etc into your determination of whether or not a client requires 24 hour supervision?

7) If a client has multiple injuries / illnesses (psychosocial, orthopaedic, & neurological) are there scenarios where the combination of all of the above led you either to decide that 24 hour supervision is required? Or situations where it is sub threshold?

8) Did the possibility of litigation influence your clinical decision making process regarding 24 hour supervision? (have you ever experienced pressure from either defence or legal counsel, insurers, etc.) to alter your decision or influence your decision (remind of confidential nature of answers, etc.). Please describe the situation if you feel comfortable.

9) Are there any other scenarios not mentioned that you think would help us understand the clinical reasoning around 24 hour supervision?

10) What future research in regard to 24 hour supervision would you like to see?
    a. What suggestions do you have for future research?
